# Movement Patterns of Juvenile Whale Sharks Tagged at an Aggregation Site in the Red Sea

**DOI:** 10.1371/journal.pone.0103536

**Published:** 2014-07-30

**Authors:** Michael L. Berumen, Camrin D. Braun, Jesse E. M. Cochran, Gregory B. Skomal, Simon R. Thorrold

**Affiliations:** 1 Red Sea Research Center, King Abdullah University of Science and Technology, Thuwal, Kingdom of Saudi Arabia; 2 Biology Department, Woods Hole Oceanographic Institution, Woods Hole, Massachusetts, United States of America; 3 Massachusetts Division of Marine Fisheries, New Bedford, Massachusetts, United States of America; Institut Pluridisciplinaire Hubert Curien, France

## Abstract

Conservation efforts aimed at the whale shark, *Rhincodon typus*, remain limited by a lack of basic information on most aspects of its ecology, including global population structure, population sizes and movement patterns. Here we report on the movements of 47 Red Sea whale sharks fitted with three types of satellite transmitting tags from 2009–2011. Most of these sharks were tagged at a single aggregation site near Al-Lith, on the central coast of the Saudi Arabian Red Sea. Individuals encountered at this site were all juveniles based on size estimates ranging from 2.5–7 m total length with a sex ratio of approximately 1∶1. All other known aggregation sites for juvenile whale sharks are dominated by males. Results from tagging efforts showed that most individuals remained in the southern Red Sea and that some sharks returned to the same location in subsequent years. Diving data were recorded by 37 tags, revealing frequent deep dives to at least 500 m and as deep as 1360 m. The unique temperature-depth profiles of the Red Sea confirmed that several whale sharks moved out of the Red Sea while tagged. The wide-ranging horizontal movements of these individuals highlight the need for multinational, cooperative efforts to conserve *R. typus* populations in the Red Sea and Indian Ocean.

## Introduction

Whale sharks, *Rhincodon typus* Smith 1828, are broadly distributed throughout tropical and sub-tropical waters of the world’s oceans. Basic information is lacking about most aspects of *R. typus* life history, including growth, age at sexual maturity, pupping locations, and migration patterns. Whale sharks are observed only rarely throughout their range except for the few locations where seasonal aggregations of whale sharks occur including the Seychelles [1], western Australia [2], Belize [3] and Holbox Island on the Caribbean coast of Mexico [4]. To date, 12 whale shark aggregation sites have been identified globally [5], [6]. These local aggregations have been associated with periods of high food availability from coral or fish spawning events or plankton blooms [3], [7]–[9]. Whale shark diets vary seasonally and geographically, but they are thought to feed mainly on zooplankton as well as algae, small fishes, fish eggs, cephalopods, and other nektonic prey [8], [10]–[15].

Whale sharks were listed as “vulnerable” by the International Union for Conservation of Nature in 2000. This designation was followed by legal protection in many nations including the Maldives, the Philippines, India, Thailand, Honduras, Taiwan, and Belize [16]–[18]. However, *R. typus* are still taken in fisheries throughout most of its range either as the result of targeted fisheries or as bycatch (e.g., [19], [20]). Small harpoon and entanglement fisheries have existed for whale sharks in various regions of the world, including India, Taiwan, the Philippines, the Maldives, and Pakistan. Declining catches in the absence of evidence for reduced fishing effort suggests that at least some whale shark populations are overexploited in India [21], the Philippines [22] and Taiwan [17], [23]. Recently, whale sharks have been found to be much more valuable alive as targets for ecotourism than killed in fisheries (i.e., [10], [24]). As a result, directed whale shark harvests have decreased in some areas [17], [21], [22]. Nonetheless concerns remain that significant fisheries still threaten at least some populations [25].

Population assessments for *R. typus* have been hindered by ocean basin-scale migrations of individuals with documented movements of up to 13,000 km [26], [27]. Recent estimates based on effective population sizes calculated from genetic analyses suggest a global population of between 27,000 and 476,000 adults [28], [29]. The same analyses noted very little genetic difference among *R. typus* in the Atlantic, Indian, and Pacific Oceans, suggesting that inter-ocean movements of *R. typus* have occurred at least on evolutionary time scales [29]. More recent analyses by Vignaud et al. [30] found evidence of structure between the Atlantic and Indo-Pacific populations, but very little evidence of genetic variation within the Indo-Pacific. In any case, the degree of migratory connectivity of whale sharks on ecological time scales relevant for conservation remains unknown from most parts of the world [27]. Improving our understanding of whale shark movements is critical if we hope to have effective management and conservation for the species [31].

In this study, we identify the first seasonal aggregation site of whale sharks in the Red Sea. We report on the movements of 47 whale sharks tagged with several types of satellite transmitting tags. The tagging program identified the seasonal presence of *R. typus* at a single location in the Saudi Arabian Red Sea. The location is the first such aggregation site described from the Red Sea and represents potentially important juvenile habitat for *R. typus* populations throughout the Indian and Pacific Oceans.

## Methods

### Ethics statement

This research was carried out under the general auspices of King Abdullah University of Science and Technology’s (KAUST) arrangements for marine research with the Saudi Arabian Coast Guard and the Saudi Arabian Presidency of Meteorology and Environment. These are the relevant Saudi Arabian authorities governing all sea-going research actions in the Saudi marine environment. KAUST has negotiated a general and broad permission for marine research in Saudi Arabian Red Sea waters with these two agencies and thus there is no permit number to provide. The animal use protocol was performed in accordance with Woods Hole Oceanographic Institution’s Animal Care and Use Committee protocol #16518 and approved by KAUST’s Biosafety and Ethics Committee (KAUST does not provide a specific approval number).

### Study area

**Figure 1:**
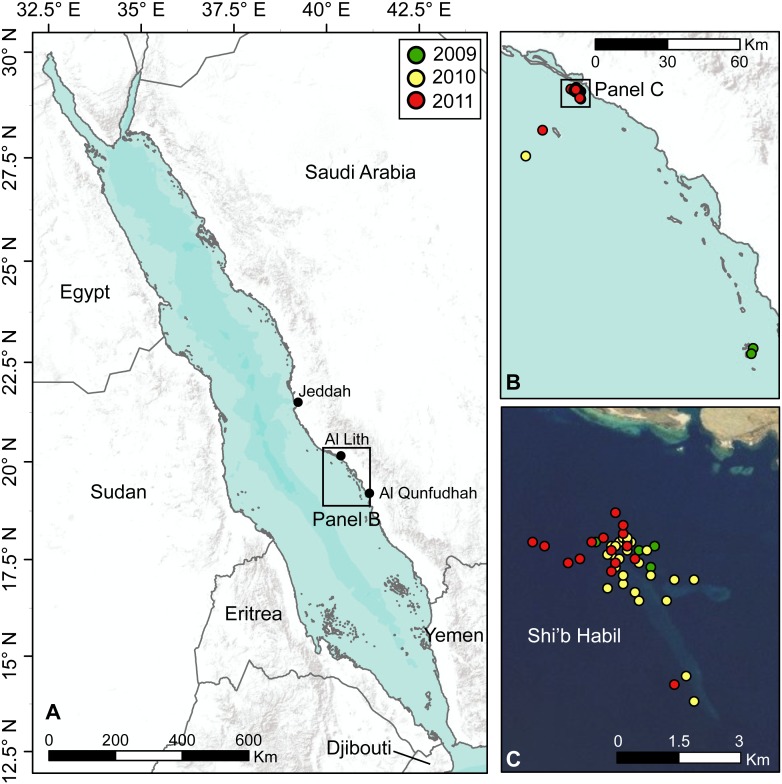
Study sites for *Rhincodon typus* in the Saudi Arabian Red Sea. (A) Location of the study area within the Red Sea. (B) Locations of 59 satellite tag deployments on juvenile *R. typus* near Al-Qunfudhah (n = 2) and Al-Lith (n = 57). (C) Detail of tag deployments around Shi’b Habil near Al-Lith (n = 55). Symbol color indicates the year of tag deployment. Basemap sources: ESRI, AND, USGS, TANA.

Reports of sporadic sightings of whale sharks from a local dive operator led us to initiate a whale shark tagging study in waters adjacent to the town of Al-Lith, ∼200 km south of Jeddah along the coast of the Saudi Arabian Red Sea (Fig. 1). The area contains numerous coral reefs on the continental shelf that extends approximately 20 km from the coast. Most of our efforts were concentrated at the northern end of Shi’b Habil, a submerged reef platform 4 km off the coast of Al-Lith. The dive boat captains reported seeing whale sharks occasionally in spring months (April to June) as they navigated past Shi’b Habil en route to popular dive sites further offshore. Opportunistic encounters with whale sharks also occurred in offshore waters 20–30 km from Shi’b Habil, and 8 km off the coast of the town of Al Qunfudhah, a further 140 km south of Al-Lith.

### Tagging

We opportunistically deployed satellite tags on whale sharks between 2009 and 2012 (Table 1). Whenever possible, the same general tagging procedures were followed. Surface-feeding whale sharks were visually located from an 11 m boat and then approached slowly. Freedivers entered the water from the vessel, estimated total length of each animal to the nearest 0.5 m, visually inspected the pelvic fin region to determine sex where possible, and took digital images for photo-identification. Finally, a satellite tag tethered to an intramuscular titanium dart was applied at the base of the dorsal fin using a sling spear.

**Table 1:** Summary information from satellite tag deployments on *Rhincodon typus* in the Saudi Arabian Red Sea.

Whale SharkPTT	Tag Type	Tag Date	Tag Lat(°N)	Tag Long(°E)	Est. Length (m)	Sex	Pop-off Date	PopLat (°N)	PopLong (°E)	DeployDuration (days)	MaxDepth (m)	TrackDistance (km)	Fig. 3	GeolocationMethods
93897	Mk10	3/29/09	20.125	40.218	5	F	4/9/09	19.550	40.780	11	360	86	A	T,B
93899	Mk10	3/28/09	20.129	40.215	3		2/6/10	20.050	40.410	315	984	22	A	T,B
93900	Mk10	3/29/09	20.130	40.219	4		2/4/10	18.720	37.598	312	416	315	A	T,B
93901	Mk10	3/29/09	20.131	40.204			DNR							
95971	Mk10	6/13/09	19.130	40.940	4	F	1/5/10	18.370	41.060	206	840	86	A	T,B
95972	Mk10	6/13/09	19.130	40.940	4	F	3/5/10	13.530	42.550	265	480	675	A	T,B
52528	Mk10	4/12/10	20.131	40.213	5	M	9/30/10	13.348	57.969	171	1184	2580	C	T,B
52529	Mk10	4/15/10	20.117	40.222	6	M	DNR							
52535	Mk10	4/4/10	20.129	40.217	3		9/30/10	17.255	41.580	179	344	370	A	T,B
52536	Mk10	4/16/10	20.128	40.208	7	F	DNR							
52537	Mk10	4/15/10	20.122	40.229	5	F	10/22/10	23.977	37.200	190	736	580	B	T,B
52538	Mk10	4/12/10	20.125	40.209	4	F	10/26/10	16.885	42.421	197	352	450	A	T,B
52539	Mk10	4/12/10	20.126	40.215	3	F	10/6/10	10.544	45.198	177	536	1250	C	T,B
52555	Mk10	3/30/10	20.122	40.224			7/10/10	26.694	36.087	102	656	920	B	T,B
52557	Mk10	4/12/10	20.129	40.212	3.5	M	1/2/11	15.299	40.471	265	184	520	A	R,T
52561	Mk10	4/12/10	20.099	40.227	4	F	5/19/10	17.458	41.194	37	344	330	A	T,B
52562	Mk10	3/29/10	20.122	40.224			6/29/10	16.286	40.662	92	760	450	A	T,B
52563	Mk10	3/29/10	20.122	40.224	4		DNR							
52565	Mk10	3/29/10	20.122	40.224	3	F	DNR							
52569	Mk10	3/29/10	20.122	40.224	5		DNR							
52570	Mk10	5/4/10	20.131	40.210	3	F	10/13/10	19.235	38.521	162	696	210	A	T,B
52571	Mk10	4/12/10	20.121	40.211	5	M	12/31/10	17.359	39.663	263	416	330	A	R,T,B
52579	Mk10	4/15/10	20.130	40.208	2.5	M	DNR							
52581	Mk10	5/4/10	20.093	40.229	3.5	M	10/8/10	19.865	40.585	157	352	50	A	T,B
52584	Mk10	4/16/10	20.119	40.214	6	F	12/31/10	18.743	38.325	259	576	265	A	T,B
52585	Mk10	3/30/10	20.122	40.224			9/30/10	11.387	49.304	184	848	1640	C	T,B
52588	Mk10	4/16/10	20.119	40.214	3		9/30/10	13.698	49.914	167	984	1700	C	R,T,B
52589	Mk10	4/16/10	20.123	40.211	3	M	DNR							
52590	Mk10	4/15/10	20.132	40.211	4	F	DNR							
52593	Mk10	5/4/10	20.117	40.215	3	M	11/13/10	19.990	37.701	193	736	285	A	T,B
52595	SPOT5	4/22/10	20.132	40.212	3.5	M	5/9/10	19.826	40.522	17	NA	50	A	R,A
52596	SPOT5	3/29/10	20.122	40.224	4		9/1/10	20.008	40.452	156	NA	30	A	R,A
52598	SPOT5	4/22/10	20.130	40.209	3	M	8/18/10	15.892	41.506	118	NA	520	A	R,A
52616	SPOT5	3/28/10	20.123	40.218	4		4/19/10	20.106	40.258	22	NA	5	A	A
52617	SPOT5	3/28/10	20.128	40.207	4.5		4/13/10	19.961	40.492	16	NA	40	A	R,A
52618	SPOT5	3/27/10	20.127	40.210	3.5		7/8/10	18.301	59.923	103	NA	2950	C	R,A
52619	SPOT5	4/22/10	20.131	40.212	7	M	7/13/10	13.924	41.803	82	NA	750	A	R,A
52620	SPOT5	4/22/10	20.132	40.212	3	M	7/31/10	14.031	42.709	100	NA	760	A	R,A
52621	SPOT5	3/28/10	20.120	40.207	4.5		8/23/10	18.660	39.492	148	NA	210	A	R,A
52622	SPOT5	4/4/10	19.873	40.002	4		7/28/10	22.859	38.845	115	NA	380	B	A,P
106744	Mk10	3/31/11	20.097	40.224			10/1/11	16.140	41.026	184	568	470	A	T,B
106745	Mk10-AF	4/2/11	20.138	40.209		M	10/1/11	18.711	40.438	182	1360	180	A	T,B
106746	Mk10	4/7/11	20.127	40.214	4	F	10/16/11	17.211	41.144	192	536	360	A	T,B
106747	Mk10-AF	3/31/11	20.130	40.212		F	6/7/11	20.155	40.298	68	526	10	A	R,T,B
106748	Mk10-AF	3/31/11	20.133	40.211	4	M	DNR							
106749	Mk10	4/2/11	20.135	40.211	4	F	DNR							
106750	Mk10-AF	4/14/11	20.097	40.224			10/3/11	15.904	41.130	172	144	500	A	T,B
106751	Mk10-AF	3/31/11	19.973	40.071	3	F	9/22/11	15.084	42.099	175	296	610	A	R,T
106752	Mk10-AF	4/4/11	20.132	40.206	4.5	F	1/10/12	18.504	39.038	281	1096	235	A	T,B
106753	Mk10	4/19/11	20.126	40.209	3		6/29/11	21.931	38.867	71	456	283	A	A
106754	Mk10-AF	4/19/11	20.124	40.208	3.5	M	7/27/11	16.266	40.556	99	584	450	A	R,A
106755	Mk10-AF	4/18/11	20.097	40.224		F	10/21/11	16.257	40.366	186	352	515	A	F,A
106756	Mk10-AF	4/19/11	20.097	40.224	3.5	F	10/1/11	16.664	41.074	165	504	420	A	F,A,T,B
106757	Mk10-AF	4/20/11	20.126	40.197	4.5	M	5/15/11	19.606	40.692	25	432	83	A	F,A
106761	Mk10-AF	4/18/11	20.130	40.191		M	7/11/11	15.082	41.248	84	472	530	A	T,B
106762	Mk10-AF	4/18/11	20.131	40.188	4	M	6/27/11	19.576	40.254	70	584	65	A	F,R,T
106763	Mk10-AF	4/17/11	20.127	40.200	3		6/17/11	17.533	41.349	61	528	325	A	R,T
106764	Mk10-AF	4/19/11	20.131	40.203	3.5	F	7/6/11	19.857	39.767	78	360	59	A	F,A
106774	Mk10	3/30/11	20.129	40.208	5	F	DNR							

Platform terminal transmitter (PTT) number for each tag is shown along with the model of each tag. All tags were manufactured by Wildlife Computers, Inc. (WA, USA). Tag Date = date of tag deployment; Tag Lat/Long = GPS coordinates of tag deployment; Est. Length = the total length (m) of the individual tagged estimated by snorkelers *in-situ*; Sex = male (M) or female (F) where determination was possible by visual observation of presence or absence of claspers between the pelvic fins, no entry indicates that sex could not be confidently determined; Pop-off Date = date of tag detachment from shark; Pop Lat/Long = GPS coordinates of tag detachment location; Deploy Duration = number of days between tag deployment and detachment; Max Depth = the deepest depth (m) reported by the tag during the deployment; Track Distance = shortest straight-line distance from tag deployment to detachment location (or from tag deployment to final location for SPOT5 tags) without crossing land; Fig. 3 = the corresponding panel of Figure 3 in which a given shark’s track is plotted; Geolocation Methods = methods used to reconstruct most likely track for each tagged animal: A = Argos location, B = bathymetric correction, F = Fastloc GPS, R = shark resighted, T = Trackit model.

### Geolocation Techniques

#### Tag types

Three types of satellite tags were deployed on whale sharks (Table 1). Towed tags fitted with an Argos transmitter (Model SPOT5, Wildlife Computers, Inc., WA, USA) were used to track individual sharks using standard Doppler-based geolocation. These tags did not have archival capabilities. Pop-up satellite archival transmitting (PSAT) tags (Models Mk10-PAT and Mk10-AF; Wildlife Computers, Inc., WA, USA) logged depth, temperature, and light level data every 10 (Mk10-AF) or 15 (Mk10-PAT) seconds to onboard memory. Archived data were compiled every 12 hours into 14 depth and 14 temperature bins that varied little among tag types. Tags also recorded a summarized temperature-depth profile every 12 (Mk10-PAT) or 24 (Mk10-AF) hours. In addition, the Mk10-AF housed a Fastloc global positioning system (GPS) transmitter for acquiring location information. After detachment, the pre-processed archived data were transmitted and retrieved through the Argos satellite system.

#### Track Reconstruction

A combination of techniques was used to estimate the most probable track for a given individual based on the type, amount, and quality of data acquired from the shark’s tag (Table 1). All tags acquired location estimates from Argos satellites while at the surface, and each location was assigned a corresponding error class (Z, B, A, 0, 1, 2, 3) with accuracy estimates to within 150 m (class 3). All locations with accuracy class Z and all locations reported from above sea level were eliminated. We also eliminated all B error class locations if the position was conspicuously erroneous based on prior and subsequent locations of higher accuracy. Tracks for SPOT5-tagged individuals were built using the Argos positions remaining after the above filtering method.

Light-level data archived and transmitted from the PSAT tags were used for light-based geolocation. Customized routines for R [32] were used to parse light, temperature, and depth data [33] for track reconstruction using the trackit R library [34], [35]. ETopo 2-minute bathymetry (http://www.ngdc.noaa.gov/mgg/global/etopo2.html) was used for the bathymetric correction according to methods in the analyzepsat package for R [33]. Positions based on individual photo identification were also used when sharks were opportunistically resighted.

Areas of core whale shark activity were determined from available location estimates for each individual as described above (Table 1). If multiple locations were acquired in a single day, positions were averaged to generate daily location estimates. Probability density was calculated per 0.05° cell covering the Red Sea basin (10–30°N, 30–50°E) and converted to a volume [36]. These probability densities were then used to generate seasonal distributions to identify variability in high-use areas throughout the year using the GenKern package for R [37] and custom functions included in the analyzepsat package [33]. Seasons were defined according to the lunar calendar.

## Results

**Figure 2:**
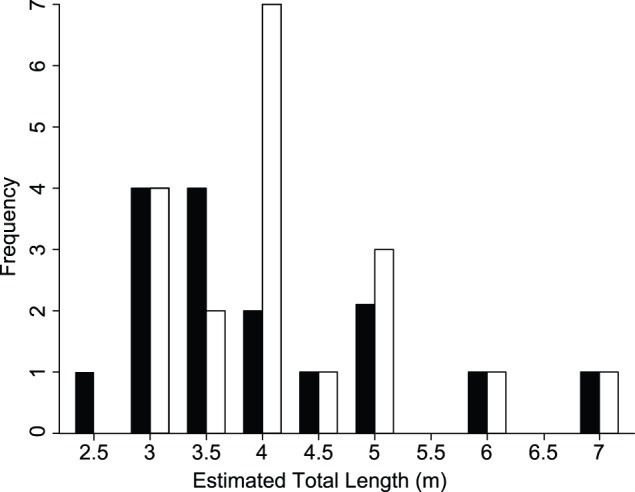
Size frequency histogram of *Rhincodon typus* individuals of known sex tagged with satellite tags at an aggregation site in the Saudi Arabian Red Sea. Bars represent the number of individuals estimated to the nearest 50

We deployed 59 satellite tags on 57 unique individual whale sharks (Table 1). Almost all tags (55 of 59) over the three years were deployed on individuals in the vicinity of Shi’b Habil (Fig. 1c). A further two sharks were tagged 20–30 km offshore of Al-Lith in 2010, and two individuals were tagged at the same location approximately 120 km south of Al-Lith and 8 km off the coast. Usable identification images for 52 sharks were submitted to www.whaleshark.org. Several sharks sighted after 2009 had PSAT tag tethers, presumably from our satellite tags deployed in earlier years. Two such sharks tagged in 2009 were confirmed to have been re-tagged in a subsequent year (one in 2010 and one in 2011) based on photo identification and the presence of old tag tethers. We therefore concluded that 59 tags were deployed on 57 individuals. Estimated sizes of tagged sharks ranged from 2.5–7.0 m, with a mean total length (TL) of 4.0 m (±0.15 m SE). We tagged 21 female sharks (mean TL 4.26 m±0.3 m SE) and 18 male sharks (mean TL 4.00 m±0.3 m) (Fig. 2), with a resulting sex ratio (M:F) of 1.06 (the 18 remaining sharks were of undetermined sex).

### Satellite tags

**Figure 3:**
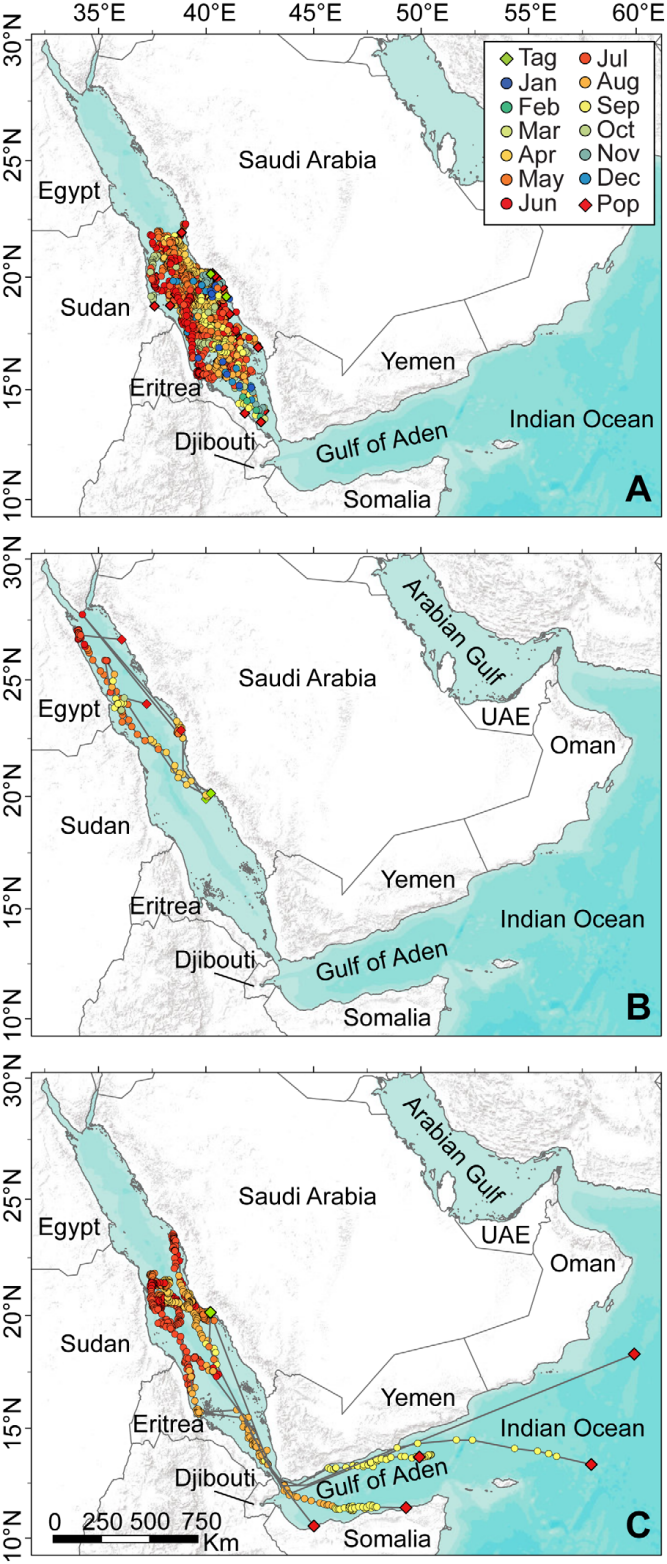
Movements of 47 *Rhincodon typus* tagged with satellite tags in the Saudi Arabian Red Sea. (A) Most individuals (n = 39) made basin-scale movements within the southern Red Sea. (B) Three *R. typus* performed excursions into the northern Red Sea as far as Sharm el-Sheikh. (C) Five sharks departed the Red Sea and moved into the Gulf of Aden and northern Indian Ocean. Green and red diamonds indicate tagging and tag pop-off locations, respectively. Track lines were removed from (A) for clarity. Basemap sources: ESRI, AND, USGS, TANA.

We received data from 47 of 59 tags deployed for 11–315 days between 2009–2012 (Table 1). The majority of tags popped up (PSATs) or stopped transmitting (SPOTs) in the southern Red Sea after exhibiting regionally-restricted movements (n = 39, Fig. 3A). However, three individuals moved north from the tagging location as far as Sharm el-Sheikh, Egypt (Fig. 3B). The remaining five individuals departed the Red Sea, moved into the Gulf of Aden, and continued as far as the northern Indian Ocean off the Omani coast (Fig. 3C). Based on Argos positions at tag release, individuals travelled up to 2950 km (shortest oceanic straight-line distance from tagging to final known location) during tag deployments. The two individuals tagged near Al Qunfudhah showed similar movement patterns when compared to those tagged near Al-Lith. The five sharks that left the Red Sea consisted of one male individual and four individuals of unknown sex. The size range for these sharks ranged from 3 to 5 m at the time of tagging (Table 1). The deployment duration for all of these tags was ∼180 days (Table 1). We are therefore unable to identify any differences between the individuals that remained in the Red Sea and the individuals that ventured into the Indian Ocean.

**Figure 4:**
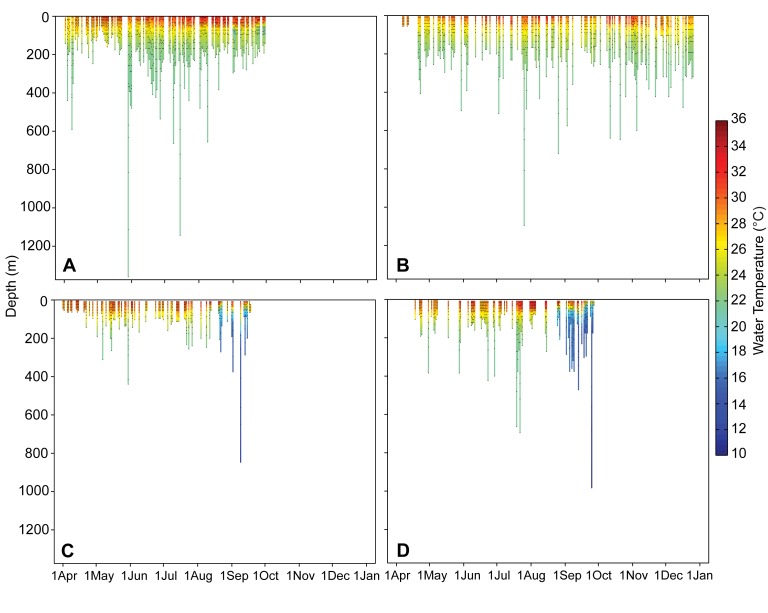
Daily depth-temperature plots for four *Rhincodon typus* tagged near Al-Lith in the Saudi Arabian Red Sea. Depth is indicated on the y-axis and by the length of the colored data column. Time is indicated on the x-axis with each data column representing a day of reported data. Days without a column indicate that no data were received for that day. Water temperate at a given depth is indicated by the color of the column (temperature scale in °C indicated on the right-hand axis). (A) Platform Terminal Transmission ID 106745, a male shark of unrecorded length tagged in April 2011. (B) PTT ID 106752, a 4.5 m female shark tagged in April 2011. (C) PTT ID 52585, a shark tagged in March 2010. (D) PTT ID 52588, a 3 m shark tagged in April 2010.

Tagged whale sharks regularly dove to 400 m. Three individuals made excursions below 1000 m, with a maximum recorded depth of 1360 m (Fig. 4). Tags recorded water temperatures ranging from 34°C at the surface to a minimum of 8°C. Notably, 21.7°C was the minimum temperature experienced by all individuals year-round at water depths below 200 m in the Red Sea. Temperatures below 21.7°C could only be recorded by tags outside of the Red Sea, thus confirming departure from the Red Sea for these PSAT-tagged sharks (Fig. 4C, D).

**Figure 5:**
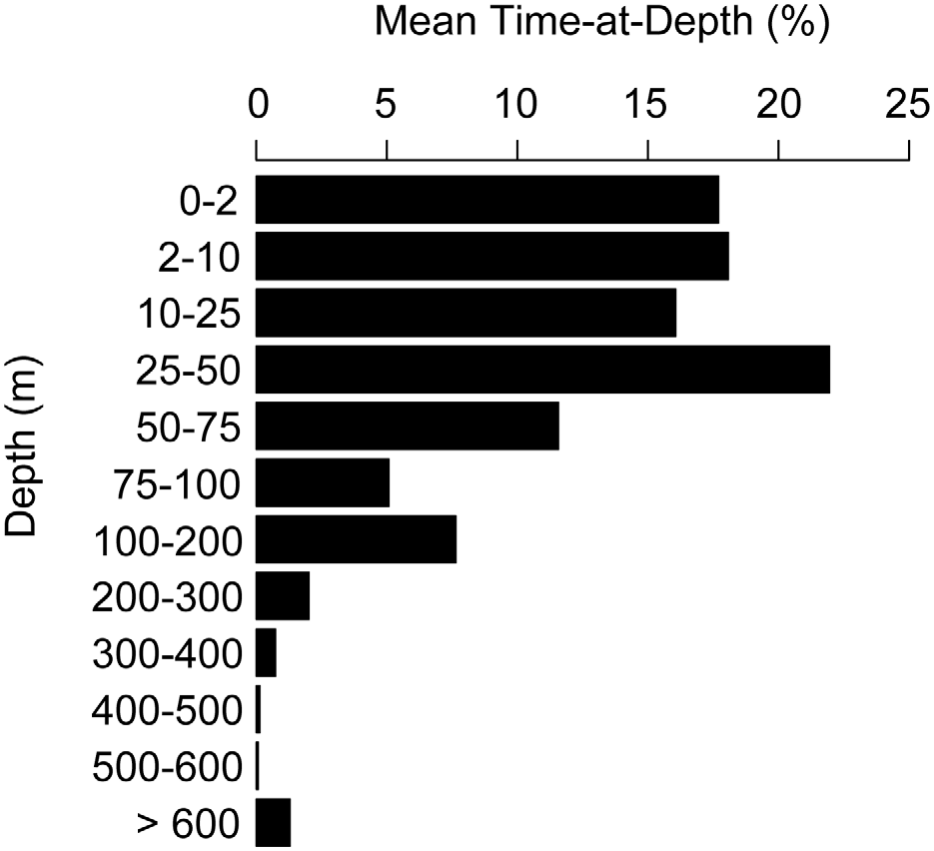
Composite time-at-depth histogram for 32 *Rhincodon typus* tagged with pop-up satellite archival tags in the Saudi Arabian Red Sea in 2010–2011. (Sharks tagged in 2009 were excluded from this analysis due to low bin resolution in transmitted time-at-depth data.) Data in horizontal bars represent the reported mean time spent in a particular depth range by individuals over the course of tag deployment. Note variable depth intervals on y-axis.

**Figure 6:**
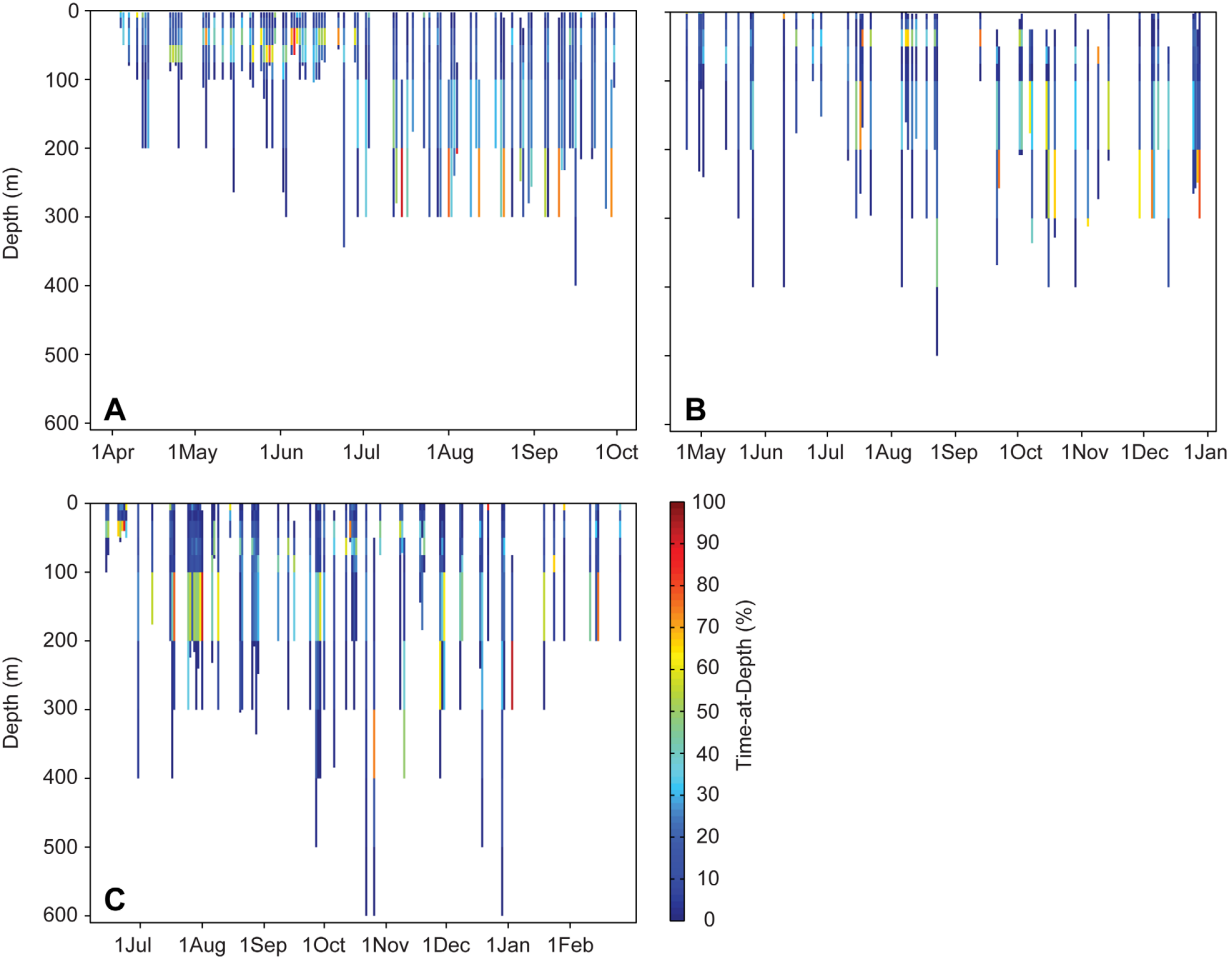
Time-at-depth plots for three *Rhincodon typus* tagged near Al-Lith in the Saudi Arabian Red Sea that exhibit considerable occupation of deep water. Depth is indicated on the y-axis and by the length of the colored data column. Time is indicated on the x-axis with each data column representing a day of reported data. Days without a column indicate that no data were received for that day. The percentage of time spent within a given depth range on a given day is indicated by the color of the column (percentage scale indicated on the right-hand axis adjacent to panel C). (A) Platform Terminal Transmission ID 52535, a 3 m shark tagged in April 2010. (B) PTT ID 52571, a 5 m male shark tagged in April 2010. (C) PTT ID 95972, a 4 m female tagged in June 2009. Note variable scale of x-axis.

Tagged sharks spent the majority of their time in the upper 50 m (Fig. 5), but occasionally spent up to 80% between 200–400 m during a 24-hour period (Fig. 6). Despite frequent occupation of the upper layers, however, sharks spent remarkably little time at the surface-air interface. The 32 sharks with reporting PSAT tags deployed in 2010–2011 spent only 16.7% of their time in the top 2 m. Note that sharks tagged in 2009 are excluded from this analysis due to a lower bin resolution in the transmitted time-at-depth tag data.

**Figure 7:**
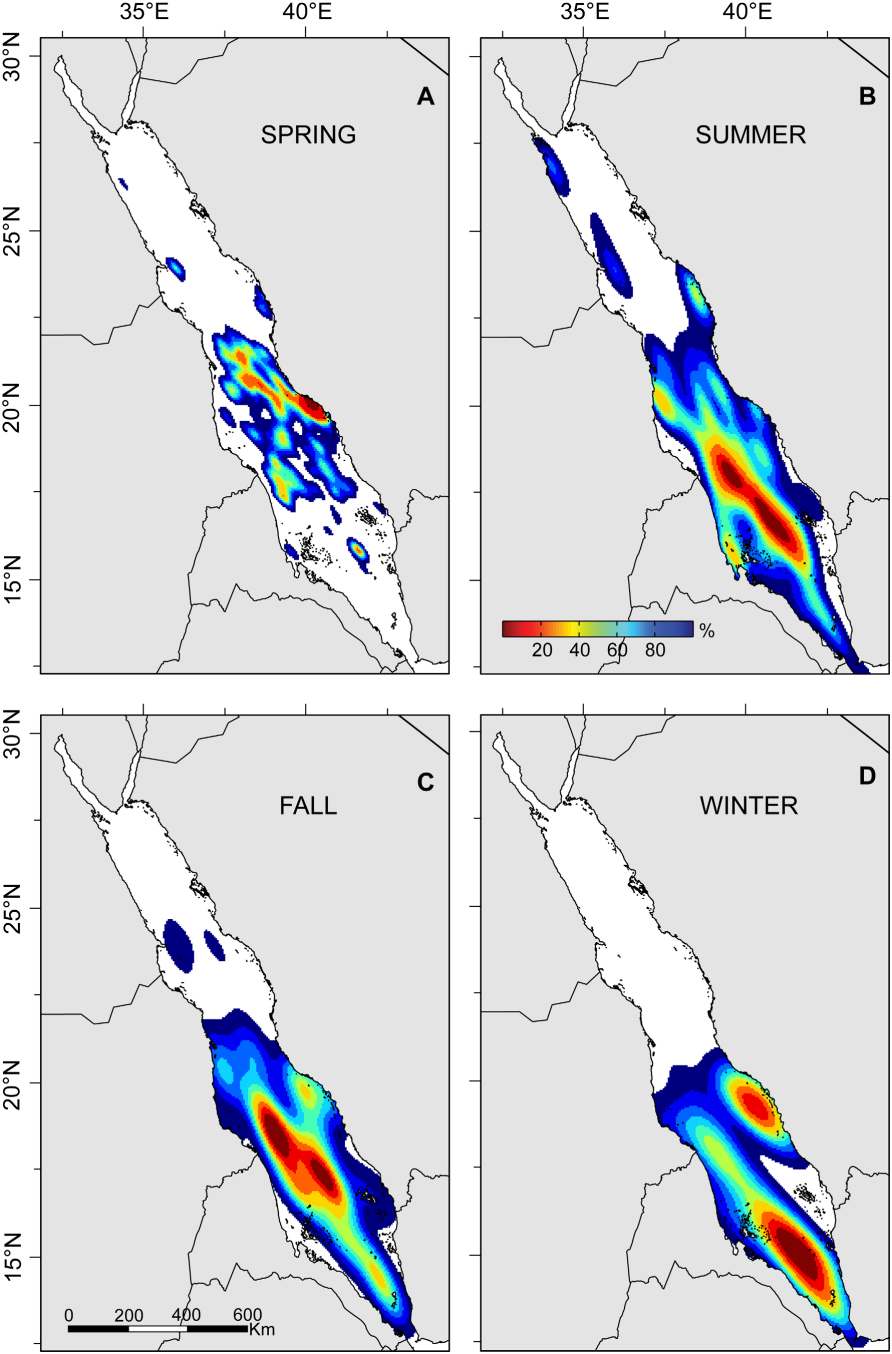
Habitat utilization distribution (UD) aggregated for all 47 whale sharks tagged with pop-up satellite archival transmitting (PSAT) tags in the Saudi Arabian Red Sea in 2009–2011. Seasons were defined according to lunar calendar. UD is composed of all track locations based on methods indicated in Table 1. The overall distribution indicates core-use areas (warm colors) near Al-Lith in the spring and further offshore and southward through the remaining seasons. Color terminates at 95% UD (peripheral-use areas).

An analysis of the horizontal distribution pattern of tagged sharks revealed seasonal movements throughout much of the southern Red Sea (Fig. 7). Areas of high use include the coasts of Sudan, Eritrea, and Yemen through summer, fall, and winter, respectively. Spring distributions were strongly focused near the tagging location. However, no tags were retained into the next spring following tagging and this may skew results for large-scale habitat use during that season.

We conducted a Kolmogorov-Smirnov pairwise comparison test to assess potential differences in percent time-at-depth for tagged sharks (excluding the 2009 sharks, for which time-at-depth data were collected in different depth bins). We found no differences in vertical or horizontal movements based on size (p>0.87 for all 5 possible pairwise comparisons using length size bins of 3, 4, 5, and 6 m) or sex (p = 0.999).

## Discussion

A number of studies have tracked whale sharks using satellite archival tags throughout the world’s oceans. To date, 12 papers document a total of 69 individual whale sharks tracked using tagging technology suitable for measuring long-distance movements (Table 2). Long-distance movements of *R. typus* have been documented from the Pacific [26], the Indian [37], and the Atlantic Oceans [38]. We have added significantly to this global whale shark database, identifying a new aggregation site in the southern Red Sea and providing tracks for 47 individual sharks from satellite archival tags. Approximately 10% of the sharks we tagged left the Red Sea, suggesting that there is potentially important connectivity with whale shark populations in the western Indian Ocean (e.g., Djibouti, India, and the Seychelles). These movements may be motivated by abundant food availability associated with seasonal upwelling in the northern Indian Ocean. In contrast, very few of the sharks seemed to use the northern Red Sea. Southern Red Sea waters are generally more productive [39] and may thus be more attractive for whale sharks.

**Table 2:** Studies to date describing satellite tagging efforts to understand large-scale movements of *Rhincodon typus*.

Citation	Tag Site	Sex Ratio(M:F:U)	No. IndividualsTagged	No. TracksPublished	Duration (days) ofPublished Tracks
Eckert and Stewart 2001 [26]	Sea of Cortez, Mexico	0∶7∶8	15	11	1–1144
Eckert *et al.* 2002 [56]	Malaysia, Philippines, Luzon	DNR	6	5	3–121
Wilson *et al.* 2006 [55]	Ningaloo Reef, Australia	1∶7∶2	10	6	57–216
Hsu *et al.* 2007 [57]	Taiwan	3∶0∶0	3	3	108–208
Rowat and Gore 2007 [37]	Seychelles	1∶0∶2	3	3	19–60
Gifford *et al.* 2007 [58]	South Africa, Honduras	4∶1∶0	5	5	2–132
Wilson *et al.* 2007 [59]	Ningaloo Reef, Australia	1∶0∶0	1	1	147
Brunnschweiler *et al.* 2009 [60]	Mozambique	1∶1∶0	2	1	87
Sleeman *et al.* 2010 [61]	Ningaloo Reef, Australia	2∶3∶2	7	7	DNR
Wang *et al.* 2012 [62]	Hainan	1∶0∶0	1	1	74
Hueter *et al.* 2013 [38]	Gulf of Mexico	12∶22∶1	35	22	2–190
Hearn *et al.* 2013 [48]	Galapagos	0∶4∶0	4	4	31–167
This study	Red Sea, Saudi Arabia	18∶21∶18	57	47	11–315

Note that “No. Tracks Published” reflects only the tracks that presented movement data (*cf*. Sequeira *et al.* 2013). *DNR* = did not report.

The diving behaviors of the Red Sea whale sharks provide evidence that *R. typus* may rely at least to some degree on prey items from depths below the euphotic zone (e.g., [14], [37], [40]). Whale sharks are therefore able to access deeper habitats but may experience physiological limitations. There is, indeed, evidence of thermoregulatory behavior following dives in whale sharks [41]. The unique temperature-depth profile in the Red Sea, where temperatures remain at 21.7°C from approximately 200 m to depths >3000 m, may facilitate extended periods of deep foraging without temperature constraints. Given relatively low oxygen concentrations at depth in the Red Sea (<2 mg/l below 200 m), the whale sharks diving in these layers may become oxygen-limited. Deep-water oxygen minima have previously been suggested as a factor limiting whale shark diving depths [40].

All tagging efforts were based on whale shark sightings from the surface. Given the infrequent occupation of the surface-air interface exhibited by PSAT-tagged individuals in this study, we may have only observed a small fraction of the whale sharks present in the study area. This data suggested that a surface-based observational approach may lead to underestimates of whale shark populations (see also [5]). In addition, the low proportion of time spent at the surface further supports hypotheses that suggest surface feeding does not represent the entirety of whale shark foraging behavior (e.g., [12], [37], [40]).

The number and size of sharks observed around Shi’b Habil indicates that this location is a previously undescribed aggregation site for *R. typus*, increasing the global number of such locations to 13 [5], [6]. Adult *R. typus* were not seen in the Al-Lith site nor at the site in Djibouti, the closest aggregation to Al-Lith outside the Red Sea. It is therefore likely that both of these aggregation sites serve as “staging grounds” before these sharks move on to regional aggregations consisting of larger sharks (sensu [42]). Indeed, five of the sharks we tagged departed from the Red Sea. Based on the tracks, it appears that these individuals likely ventured further into the Indian Ocean. Three of these tags were PSATs that detached from the sharks at the end of the programmed deployments, and it appears the sharks were still in transit based on what appeared to be directed movements in the weeks leading up to detachment.

It is not known what attracts *R. typus* to Shi’b Habil. Like many other aggregation sites, it may be due to localized productivity in the area. The reef is adjacent to a very large (approximately 110 km^2^) shallow, enclosed bay largely comprised of seagrass and mangrove habitats. Individual *R. typus* are reported to associate with numerous other pelagic species such as various species of pilot fishes, mantas, and mobula rays (reviewed in [5]). At the Al-Lith site, we saw *R. typus* frequently feeding behind schools of *Atule mate* (yellowtail scad). The presence of mantas feeding in the vicinity of Shi’b Habil [43] also suggests that there may be increased productivity in this area. Yet while mantas and whale sharks co-occur at Shi’b Habil in the spring, the two species have quite different patterns of movement during the rest of the year. Mantas appear largely restricted to nearshore waters adjacent to and immediately south of Al Lith, while whale sharks disperse through the southern Red Sea. In terms of vertical distributions, both species are commonly found in the upper 100 m of the water column. However, some whale sharks both dove much deeper and spent a greater proportion of their time below 100 m compared to the mantas tagged by Braun et al. [43].

Some *R. typus* aggregations occur around feeding opportunities associated with seasonal fish or coral spawning events [3], [6], [44]. Coral spawning in the Red Sea typically occurs around full moons from April through June [45]. It is, therefore, possible that the presence of the sharks near Shi’b Habil is related to coral spawning, but this will need to be confirmed by further work. The whale sharks may be adopting a strategy to exploit several potential food resources given a potentially patchy food environment in this region. Similar coastal foraging behavior has been previously suggested by Rohner et al. [14] and Couturier et al. [15] based on data from signature fatty acid studies.

In this study, we achieved a tag reporting rate of 47 from 59 tags (79.6%) which is typical for electronic tag deployments [46]. Unfortunately, we do not know why 12 tags did not report. It is unlikely that tag attachment was a problem because a prematurely released tag would actually have been more likely to communicate with us than a late-releasing tag. Non-reporting tags were spread throughout the study, indicating that it was not a batch of tags, or specific tag rigging equipment, that led to non-reporting. We suspect that at least some of the non-reporting tags were excessively covered with biofouling and thus failed to communicate with the satellites upon detachment. It is also possible that the release mechanisms failed or were similarly biofouled in such a way that precluded detachment of the tag from the tether once the burn wire was activated.

The whale shark aggregation site near Al-Lith is unique because of the number of females that are present. Male sharks dominate all of the known aggregation sites in the Indian and western Pacific locations [5], despite neonatal *R. typus* sex ratios of approximately 1∶1 [47]. Several of the eastern Pacific aggregations are dominated by large females (e.g., southern Sea of Cortez [26] and Galapagos [47]). The presence of small, presumably immature [49] sharks at apparent sexual parity is therefore particularly intriguing and raises questions of when and why sex segregation occurs in *R. typus*. There is some evidence to suggest that females may occupy a distinct and more pelagic habitat compared to males [50]. However, exactly when this change in habitat use occurs in not known as few young-of-the-year *R. typus* have ever been found [5], [19]. It seems likely that the southern Red Sea and Djibouti serve as key juvenile habitats for populations in the Indian Ocean [42], and it is possible that other undocumented hotspots exist in the under-studied Red Sea [51]–[53]. Nonetheless, the presence of significant numbers of *R. typus* in the vicinity of Shi’b Habil indicates that the southern Red Sea should become a major regional priority for conservation efforts in the Indian Ocean. Fortunately, to our knowledge, neither Saudi Arabia nor Djibouti has any active harvesting of whale sharks. Ship strikes, however, were identified as a threat to whale sharks in the Red Sea nearly a century ago [54], and the Suez Canal currently accommodates traffic of about 17,000 vessels entering/leaving the Red Sea each year (Suez Canal Authority, www.suezcanal.gov.eg). Given evidence of population connectivity between the Red Sea and the Indian Ocean, threats to these juvenile aggregation sites could have drastic long-term effects on *R. typus* populations in the latter. The first step toward protecting these sites is to confirm and report their existence.

Whale sharks may be particularly vulnerable to exploitation due to their migratory nature. Their presumed life history traits, including long lifespan, low fecundity, delayed maturation, and slow, shallow swimming habits [2], [5], [56] also make them susceptible to directed harvest or bycatch. Other whale shark tracking studies, coupled with the results from our study, indicate that whale sharks cross many political boundaries during their lives and thus may be particularly vulnerable to inadequate management. An international conservation and management effort is necessary in order to adequately protect whale sharks from further decline [11], [17], [21], [22], [55]. Although discussion has begun with CITES (Convention on International Trade in Endangered Species) and CMS (Convention on Migratory Species), a management plan has yet to be solidified and enacted by countries with significant numbers of whale sharks in their Exclusive Economic Zones. Aside from harvest bans in some countries, little more than discussion has occurred toward managing these sharks on a regional or trans-oceanic scale.

The vast majority of studies on *R. typus* to date have focused on very few individual accounts, limiting our ability to understand species- or population-level traits critical for sound conservation. With such an unprecedented and comprehensive study of this population, we have identified several areas intensively used by whale sharks in the Red Sea. This information is critical for developing conservation and management strategies for this charismatic fish.
